# Heyde Syndrome Treated by Conventional Aortic Valve Replacement

**DOI:** 10.21470/1678-9741-2019-0004

**Published:** 2019

**Authors:** Leonardo Rufino Garcia, André Monti Garzesi, Guilherme Tripoli, Nelson Leonardo Kerdahi Leite de Campos, Antonio Sérgio Martins, Marcello Laneza Felicio

**Affiliations:** 1 Serviço de Cirurgia Cardiovascular, Hospital das Clínicas da Faculdade de Medicina de Botucatu, Universidade Estadual Paulista (UNESP), Botucatu, SP, Brazil.

**Keywords:** Aortic Valve Stenosis, Heart Valve Prosthesis, Gastrointestinal Hemorrhage, Anemia, Heart Failure

## Abstract

Heyde syndrome manifests as aortic stenosis associated with gastrointestinal bleeding. We describe the case of a 64-year-old man who came to the emergency room due to acute heart failure and intermittent gastrointestinal bleeding. Treatment involves initial correction of anemia and heart failure followed by aortic valve replacement. The prosthesis used depends on the characteristics of each patient and valve replacement allows the resolution of bleeding in most cases. Gastrointestinal bleeding in patients with aortic stenosis is associated with severity of the valve obstruction. A mechanical prosthesis was used with no recurrent bleeding even with the need for lifelong anticoagulation therapy.

**Table:** 

Abbreviations, acronyms & symbols	
ADAMTS 13	= Disintegrin and metalloproteinase with thrombospondin type 1 motif, member 13
AS	= Aortic stenosis
TAVI	= Transcatheter aortic valve implantation
vWF	= Von Willebrand factor

## INTRODUCTION

Aortic stenosis (AS) is increasingly prevalent due to increased life expectancy and the consequent population aging. In developed countries, it is the most common primary valvopathy. The prevalence of severe AS is 1-2% at the age of 75 years, rising to 6% at 85 years^[1]^. Critical AS may present with syncope, angina and dyspnea, and risk factors are similar to those for atherosclerosis^[1]^. In 1958, Heyde described an association between AS and gastrointestinal hemorrhage^[2]^, which was later studied and understood^[3]^. We present a case of Heyde syndrome treated by conventional surgery and mechanical prosthesis implantation with a satisfactory evolution.

## CASE REPORT

A trader, hypertensive, diabetic, and ex-smoker 64-year-old man, with chronic atrial fibrillation, had a history of previous stroke (occurring in January 2017), being discharged from the hospital after one month. Soon after discharge, he came to the emergency room with lower limb edema and dyspnea at rest (beginning 20 days after discharge), with progressive worsening and orthopnea. He also reported occasional episodes of melena of varying intensity.

An echocardiogram was performed, which identified a severe AS, with aortic valve presenting gross calcifications of the leaflets, reduction of mobility, valve area of 0.6 cm^2^, maximum and medium gradient of 60 and 36.5 mmHg, respectively, and ejection fraction of 67% (Teichholz method). Angiocoronography showed a 40% obstruction in the right coronary artery. As the digestive hemorrhage persisted, the patient underwent an enteroscopy that evidenced the presence of angiodysplasias in the jejunum.

Based on the clinical findings, the diagnosis of Heyde syndrome was then made. The patient received clinical treatment for acute heart failure and anemia, then he was submitted to surgery for conventional aortic valve replacement, with a mechanical 23 mm optimized double leaflet prosthesis, in January 2018. He was extubated four days after surgery. However, there was an extubation failure due to pneumonia and a tracheostomy was performed. Acute renal injury also occurred in the early days after surgery. The patient improved and was discharged from the intensive care unit on the 17^th^ postoperative day. After the surgery, there were no new episodes of digestive hemorrhage.

## DISCUSSION

Heyde syndrome is the association of AS with gastrointestinal bleeding due to gastrointestinal angiodysplasia. In a series of cases with 50 patients with severe-to-moderate AS, about 20% of them had some type of preoperative bleeding^[4]^.

The clinical presentation with digestive hemorrhage is related to the type 2A acquired von Willebrand syndrome that occurs in these patients^[5]^. The passage of von Willebrand factor (vWF) by valve stenosis causes the proteolysis of its high-molecular-weight multimers by a proteinase that acts preferentially under high shear stress situations, called ADAMTS 13 (a disintegrin and metalloproteinase with a thrombospondin type 1 motif, member 13)^[2]^. Previous studies have shown that there is an inverse relationship between the mean aortic transvalvar gradient and the percentage of high-molecular-weight multimers of vWF in the circulation^[4]^. The vWF is secreted by the endothelium and its multimers are important for adequate platelet-mediated hemostasis when they bind to platelet glycoprotein Ib^[5]^.

A possible explanation for the emergence of gastrointestinal angiodysplasias is that severe AS may be associated with a decrease in gastrointestinal perfusion with consequent dilation of blood vessels by hypoxemia, leading to fixed vasodilation, the origin of angiodysplasia^[5]^. Another theory is that mucosal hypoxia might be caused by cholesterol emboli from the aortic valve or by the altered pulse waveform in AS. Angiodysplasia have been described in hypertrophic cardiomyopathy, in which the alteration of the pulse waveform is also found^[6]^.

Angiodysplasias may occur anywhere in the gastrointestinal tract, but they are more common in the ascending colon, particularly the caecum^[6]^. A total of 1-6% of in-patient gastrointestinal bleedings are caused by angiodysplasias, while 30-40% of gastrointestinal bleedings of an obscure source are found to be linked to angiodysplasia and it is possibly the most common cause of lower gastrointestinal bleeding in the elderly^[6]^.

It is well stablished that the best treatment in such cases is aortic valve replacement. It has been shown that there is a significant increase in the percentage of the high-molecular-weight multimers of vWF in the first hours after surgery, thus limiting the risk of further hemorrhage^[4]^, a potentially serious complication in the postoperative period. However, it has been demonstrated that in the presence of prosthesis-patient disproportion, also called mismatch (valve area less than 0.85 cm^2^/m^2^ at the echocardiogram), recurrent gastrointestinal bleeding occurs due to the maintenance of the pathophysiological mechanism^[5]^. It has been proposed that guidelines for the management of AS should have bleeding from intestinal angiodysplasia with demonstrable acquired type 2A von Willebrand syndrome added as an indicator for valve replacement, and monitoring the severity of the syndrome should be considered as one of the factors determining the timing for surgery^[6,7]^.

Another point to be debated is the best type of prosthesis to be implanted. The choice should consider the characteristics of each patient. Figuinha et al.^[5]^ and many other authors affirm that the prosthesis of choice is biological in all cases of Heyde syndrome, possibly due to the reduced risks of hemorrhagic postoperative complications. However, in this case, the patient would already require anticoagulation due to chronic atrial fibrillation with previous embolic event. Even with anticoagulation therapy, that started in the second day after surgery, there was no bleeding, which might be highlighted. So, it indicates that the correct understanding of the pathophysiology positively influences the choice of treatment.

Although the surgery was uncomplicated, the morbidity was not negligible due to postoperative complications. Thus, a transcatheter aortic valve implantation (TAVI) could have been proposed. Since the first clinical report in 2002, TAVI has emerged as a valuable, less invasive and safe therapeutic alternative in patients with severe AS^[8]^, but it is not performed in the Brazilian Public Health System yet. Currently, the great advantage of TAVI over conventional valve replacement is the decrease in morbidity and mortality rates, especially for high-risk patients.

AS has a high prevalence rate today due to an increase in population life expectancy and its association with gastrointestinal bleeding, the so-called Heyde syndrome, may be more frequent than presently assumed. Therefore, physicians should be aware of this type of clinical presentation, since the presence of digestive hemorrhage may be associated with hemodynamically important valve stenosis. When choosing the type of prosthesis, cardiac surgeons must consider the special characteristics of each patient. A very different aspect in this case is that a mechanical valve was selected and there was no bleeding recurrence even with the need for lifelong anticoagulation therapy.

**Table:** 

Authors' roles & responsibilities	
LRG	Substantial contributions to the conception or design of the work; or the acquisition, analysis, or interpretation of data for the work; final approval of the version to be published
AMG	Substantial contributions to the conception or design of the work; or the acquisition, analysis, or interpretation of data for the work; final approval of the version to be published
GT	Substantial contributions to the conception or design of the work; or the acquisition, analysis, or interpretation of data for the work; final approval of the version to be published
NLKLC	Drafting the work or revising it critically for important intellectual content; final approval of the version to be published
ASM	Drafting the work or revising it critically for important intellectual content; final approval of the version to be published
MLF	Substantial contributions to the conception or design of the work; or the acquisition, analysis, or interpretation of data for the work; final approval of the version to be published
